# Idiopathic multicentric Castleman disease in children: a single-center retrospective analysis

**DOI:** 10.1186/s12887-024-05347-0

**Published:** 2025-01-17

**Authors:** Junye Du, Jiafeng Yao, Honghao Ma, Li Li, Ang Wei, Liping Zhang, Dong Wang, Zhigang Li, Rui Zhang, Tianyou Wang

**Affiliations:** 1 Hematology Center, Beijing Key Laboratory of Pediatric Hematology Oncology, National Key Discipline of Pediatrics (Capital Medical University), Key Laboratory of Major Disease in Children, Ministry of Education, Department of Hematology, Beijing Children’s Hospital, Capital Medical University, National Center for Children’s Health, Nanlishi Road No. 56, Xicheng District, Beijing, 100045 China; 2 Hematologic Disease Laboratory, Beijing Key Laboratory of Pediatric Hematology Oncology; National Key Discipline of Pediatrics (Capital Medical University); Key Laboratory of Major Disease in Children, Ministry of Education, Beijing Pediatric Research Institute; Hematology Center, Beijing Children’s Hospital, Capital Medical University, National Center for Children’s Health, Beijing, 100045 China; 3 Department of Hematology, Beijing children’s Hospital, Capital Medical University, Nanlishi Road No.56, Xicheng District of Beijing, Beijing, 100045 P.R. China

**Keywords:** Castleman disease, Pathogenesis, Treatment, Children

## Abstract

**Objective:**

To investigate the clinical features, pathological phenotype, treatment and prognosis of idiopathic multicenter Castleman disease (iMCD)in children.

**Methods:**

From January 2017 to September 2023, basic information, laboratory tests, treatment and prognosis of children diagnosed with iMCD who attended Beijing Children's Hospital of Capital Medical University were collected.

**Results:**

A total of 9 children were enrolled, with a median age of onset of median 11 (2–15) years, 6 males and 3 female. 3 cases were pathologically typed as plasma cell type, 1 case was mixed type, and the remaining 5 cases were hyaline vascular type. 9 children received different regimens of chemotherapy. The median follow-up time was 26 (13, 58) months, with no deaths, 7/9 cases showing improvement, 1/9 cases showing stable condition, and 1/9 cases showing active condition.

**Conclusion:**

Children with multicentric CD often have systemic symptoms, lymph node enlargement and related compression symptoms are the most common manifestations, followed by fever, malaise and other systemic symptoms. Anti-IL-6-based therapy combined with hormones lenalidomide and other medications have a specific therapeutic effect on multicentric CD.

## Introduction

Castleman disease (CD) is a lymphoproliferative disorder. Based on its pathological features, it can be categorized into hyaline vascular type, plasma cell type, and a mixed type. The diagnosis of CD currently mainly relies on the results of histopathological examination of the affected lymph nodes. The hyaline vascular type is characterized by aberrant germinal centers penetrated by hyaline vessels and associated with abnormal follicular dendritic cells. The plasma cell type is characterized by normal or enlarged germinal centers related with interfollicular plasma cell infiltration [1]. Clinically, it is often classified into unicentric CD (UCD) and multicentric CD (MCD) according to the distribution of the affected areas. Most UCDs only present as lymph node enlargement, while a few come with symptoms of lymph node enlargement compression/systemic symptoms or paraneoplastic pemphigus/obstructive bronchiolitis, etc. MCD involves multiple lymph node regions, often accompanied by various systemic symptoms including fever, hepatosplenomegaly, anemia/hepatic renal dysfunction/volume overload, etc. MCD is further classified into HHV-8-associated MCD (HHV8-MCD) and idiopathic MCD (iMCD) based on the presence of human herpesvirus-8 (HHV-8) infection. Previous research has shown that the incidence of UCD is much higher than that of MCD, and HHV8-MCD is even rarer. Due to the low incidence, current understanding of the clinical manifestations, pathogenesis, and treatment options of CD is still limited. Although the exact pathogenesis of CD remains unclear, previous studies have shown that IL-6 plays a crucial role in the development of CD, especially MCD [2, 3]. The median age of onset for this disease, as reported in previous studies, is approximately 34 years old. The incidence rate in children is lower than in adults. iMCD usually affects adults [4]. Although the overall survival rate of pediatric CD patients is higher, they are more likely to experience disease progression and recurrence. Current research on the characteristics of pediatric CD patients is relatively scarce, and clinical summaries of iMCD are even rarer [5, 6]. This study aims to summarize the characteristics, treatment, and prognosis of pediatric iMCD patients in our center, review and analyze past medical history to enhance medical staff's understanding of pediatric iMCD, and summarize standardized treatment protocols.

## Research subjects and methods

### Research subjects

This research is a retrospective observational study. The study objects were nine confirmed cases of idiopathic multicentric Castleman's disease in children admitted to the Beijing Children's Hospital affiliated with Capital Medical University from January 2017 to September 2023. This study complies with the Helsinki Declaration and has been approved by the Ethics Review Committee of Beijing Children's Hospital affiliated with Capital Medical University. Patient informed consent was waived.

### Research methods

#### Inclusion criteria

Criteria for the diagnosis of idiopathic multicentric Castleman's Disease:

I. Major Criteria (need both):
  1. Histopathologic lymph node features consistent with the iMCD spectrum.
  2. Enlarged lymph nodes (≥ 1 cm in short-axis diameter) in ≥ 2 lymph node stations.
II. Minor Criteria (need at least 2 of 11 criteria with at least 1 laboratory criterion):

##### Laboratory

1. Elevated CRP (> 10 mg/L) or ESR (> 15 mm/h) 2. Anemia (hemoglobin < 12.5 g/dL for males, hemoglobin < 11.5 g/dL for females) 3. Thrombocytopenia (platelet count < 150 k/μL) or thrombocytosis (platelet count > 400 k/μL) 4. Hypoalbuminemia (albumin < 3.5 g/dL) 5. Renal dysfunction (eGFR < 60mL/min/1.73m2) or proteinuria (total protein 150 mg/24h or 10 mg/100 ml) 6. Polyclonal hypergammaglobulinemia (total γ globulin or immunoglobulin G > 1700 mg/dL).

##### Clinical

1. Constitutional symptoms: night sweats, fever (> 38°C), weight loss, or fatigue (≥ 2 CTCAE lymphoma score for B-symptoms) 2. Large spleen and/or liver 3. Fluid accumulation: edema, anasarca, ascites, or pleural effusion 4. Eruptive cherry hemangiomatosis or violaceous papules 5. Lymphocytic interstitial pneumonitis.

III. Exclusion Criteria (must rule out each of the diseases that can mimic iMCD) [7].

Criteria for the diagnosis of other severe comorbidities:

According to previous literature reports, the criteria for the diagnosis of other severe comorbidities in this paper are as follows: TAFRO syndrome [8], POEMS syndrome [9], hemophagocytic lymphohistiocytosis (HLH) [10].

### Data collection and organization

The electronic medical record system was used to collect children's clinical data, including general information, initial symptoms, positive signs, imaging examination and laboratory test results, treatment plans, and follow-up situation.

### Follow-up

Outpatient follow-up was the primary method, supplemented by telephone follow-up, to continuously assess patients after discharge. Follow-up was conducted during treatment according to treatment requirements, the first follow-up visit was 3–6 months after treatment ended, and then every half year to a year. For children who had not returned to the clinic for more than a year, telephone follow-up was used.

### Treatment plan

Currently, there is no unified treatment plan for pediatric iMCD. The main treatment methods are IL-6 receptor monoclonal antibody (tocilizumab) and IL-6 antibody (siltuximab). For some children whose treatment with tocilizumab was not effective, previous studies have shown that combination chemotherapy can achieve certain effects, such as the CHOP regimen, LD (lenalidomide, dexamethasone) regimen, etc. [11, 12]. In addition, thalidomide, cyclosporine, oral corticosteroids, and sirolimus have also been reported to be used in the treatment of iMCD [11, 13]. The specific treatment conditions of the patients are detailed in Table 1.

**Table 1:** The specific treatment conditions of the patients

	Sex	Age (years)	Main point of view	Complication	Teatment	Pathological typing	Time of last follow-up	Wihdrawal time	Prognosis
Case 1	Male	8	Multiple enlarged lymph nodes for 3 years	Pneumonia, ascites	IL-6 receptor monoclonal antibody (3 courses of treatment) → methylprednisolone(4 months) → sirolimus (4 months) → melphalan in combination with cyclosporine (4 weeks) → cyclosporine orally (12 months)	Hyaline vascular type	2022–05	2021–07–21	Improve
Case 2	Male	2	Recurrent fever for 20 days, edema of the extremities for 2 weeks	HLH, renal injury, respiratory failure, hypertension, DIC, multiple plasmapheresis, anemia	CHOP regimen chemotherapy (1 course of treatment) → IL-6 receptor monoclonal antibody (8 courses of treatment)	Hyaline vascular type	2022–07–20	2020–12–20	Improve
Case 3	Male	11	Painless swollen lymph nodes in the neck	-	IL-6 receptor monoclonal antibody (12 courses of treatment)	Hyaline vascular type	2021–01–20	2021–01–20	Improve
Case 4	Male	13	Intermittent fever, diarrhea, and weakness of both lower extremities for more than 3 months, with lymph node enlargement found for 2 weeks	Anemia, hypocalcemia, renal injury, peritoneal effusion, hypothyroidism	Methylprednisolone in combination with IL-6 receptor monoclonal antibody (1course of treatment) → lenalidomide in combination with dexamethasone (12 months) → lenalidomide (12 months)	Plasma cell type	2023–07-05	2022–11	Improve
Case 5	Male	9	Intermittent fever and malaise for 20d, anemia and thrombocytopenia found for half a month	HLH, kidney injury, pulmonary embolism, pneumonia, DIC, hypertension, multiple plasma cavities, posterior lobe leukoencephalopathy, anemia	IL-6 receptor monoclonal antibody combined with methylprednisolone (2 courses of treatment) → BCD regimen combined with thalidomide (bortezomib, cyclophosphamide, dexamethasone) chemotherapy (3 courses of treatment)	Plasma cell type	2023–08–16	2022–04–23	Improve
Case 6	Male	15	Intermittent pain in both upper extremities for 2 years	Liver impairment	(outside hospital) IL-6 receptor monoclonal antibody (21 courses of treatment) → Merovia (1 month) → (our hospital)thalidomide + dexamethasone (14 months)	Hyaline vascular type	2023–01–11	Drug not yet stopped	Stable
Case 7	Female	11	Right-sided chest pain for 1 month	Hepatic impairment, renal injury	IL-6 receptor monoclonal antibody (11 courses of treatment)	Hyaline vascular type	2023–06–13	2023–05	Activity
Case 8	Female	12	Fever with multiple plasmapheresis for more than 1 month	Pneumonia, renal injury, myocardial damage, hypertension, ocular hypertension, coagulation abnormalities	IL-6 receptor monoclonal antibody (1 course of treatment) → BCD regimen chemotherapy (3 courses of treatment)	Plasma cell type	2023–09-09	Drug not yet stopped	Improve
Case 9	Female	12	Weakness for more than 1 year, abdominal distension for more than 5 months	Adhesive bowel obstruction, sinus bradycardia	(outside hospital) IL-6 monoclonal antibody (4 courses of treatment) → IL-6 receptor monoclonal antibody (11 courses of treatment)	Mixed type	2023–09-06	Drug not yet stopped	Improve

## Results

### General information and pathological classification

There were 9 cases that met the inclusion criteria, with a median age of 11 (2–15) years old, and a male to female ratio of 6:3. In terms of histological classification, there were 5 cases of hyaline vascular type, 1 mixed type, and 2 plasma cell type. All patients were diagnosed by lymph node biopsy pathology, and there were no positive cases for HHV-8. The follow-up cut-off was September 13, 2023, with a median follow-up time of 26 (13, 58) months. There were no cases of death, 7/9 cases improved, 1/9 cases were stable, and 1/9 cases were active. The total number of newly diagnosed cases of non-Hodgkin's lymphoma (NHL) in our hospital during the same period was 115, and the ratio between iMCD and NHL was 9/115.

### Clinical manifestations

The initial symptoms of the children included fever (5/9 cases), lymph node enlargement and its resulting compression symptoms (such as abdominal pain, chest pain, etc.) (6/9 cases), fatigue (3/9 cases), and changes in blood count (1/9 cases). 8 cases had systemic symptoms, including fever (5/8 cases), vomiting (2/8 cases), and multiple serous cavity effusion (3/8 cases) being more common. Other systemic symptoms included cough, fatigue, etc. The liver, spleen, and hematopoietic system were the three most frequently affected extranodal organs or systems (5/9 cases for liver, 5/9 cases for hematopoietic system, 4/9 cases for spleen). The lungs, pancreas, kidneys, thyroid, peripheral nervous system, pituitary, bones, bone marrow, muscles, and thymus could also be affected.

In terms of severe comorbidities, patient 2 had repeated fever and hepatosplenomegaly, routine blood tests indicated pancytopenia, and serum ferritin was significantly increased, so it was judged to have HLH. Patient 5 had manifestations of multiple serous cavity effusion, routine blood tests indicated pancytopenia, and acute renal failure and increased IL-6 levels in plasma were observed, leading to a diagnosis of TAFRO syndrome. This patient also had repeated fever, hepatosplenomegaly, routine blood tests indicated pancytopenia, serum ferritin levels were elevated, NK cell activity was reduced, and serum sCD25 levels were elevated, leading to a diagnosis of HLH. Patient 4 had peripheral neuropathy, blood electrophoresis results showed elevated λ light chain protein and VEGF levels, organ enlargement, multiple serous cavity effusion, and multiple biochemical tests assisting in the diagnosis of hypothyroidism, adrenal insufficiency, and other endocrine disorders, leading to a diagnosis of variant-type POEMS syndrome associated with Castleman's disease. Children's clinical information is detailed in Table 1.

### Laboratory test results

Four children had varying degrees of anemia, with hemoglobin 91.5 (61,100) g/L; four cases had abnormal platelet counts (2 cases elevated, 2 cases decreased), with a median platelet count of 319 (32,451) × 10^9^/L; four cases had elevated CRP; four cases had elevated ESR. Regarding blood biochemistry, one case had elevated liver enzymes, which was considered to be related to organ involvement of the primary disease, and one case had elevated LDH. Patients 2 and 5 had significantly elevated ferritin levels, 504.2 ng/ml and 879.4 ng/ml respectively. All 9 children completed relevant cytokine level tests, with 7 cases showing elevated IL-6 levels, median value 42.67 (2,2531.54) pg/ml, and 1 case with elevated IL-10 levels. In situ hybridization of lymph node biopsy tissue for EBER and HHV-8 were both negative. Patients 5 and 7 completed VEGF testing and both showed varying degrees of elevation. Details of auxiliary examination are shown in Table 2.

**Table 2:** Relevant auxiliary examinations of the children

	Hb (g/L) (110–160)	Plt (× 10^9^/L) (100–400)	CRP (mg/L) (< 8)	ESR (mm/h) (0–15)	ALT (U/L) (5–40)	Creatinine (umol/L) (30–104)	LDH (U/L) (110–295)	IL-6 (pg/ml) (≤ 5.4)	IL-10 (pg/ml) (≤ 12.9)	INF-γ (pg/ml) (≤ 23.1)	VEGF (pg/ml) (≤ 142)
Case1	130	108	< 5	2	13.2	18.7	204	15	15.22	1.34	
Case2	61	40	24	2	2.5	52	329	69.21	10.23	0	
Case3	130	388	< 5	7	11.6	43.8	211	2	2.61	0.31	
Case4	100	347	< 8	63	13.5	33.1	118	264.5	1.47	0	
Case5	78	32	134	51	4.3	78.4	188	5.52	3.25	2.48	241.36
Case6	118	419	151.1	88	86.6	59.8	120	42.67	< 2.44	2.72	
Case7	145	319	< 5	2	35.1	34.2	260	2.88	< 2.44	8.7	68.75
Case8	83	161	47	30	3	62	266	218.45	< 2.44	9.35	
Case9	153	451	< 10	8	32.2	34.7	227	2531.54	< 2.44	9.35	

### Treatment and prognosis

Eight out of 9 patients (Patients 1, 3, 4, 5, 6, 7, 8, and 9) first received IL-6 receptor monoclonal antibody treatment.

Patient 2, due to concurrent HLH, initially used the CHOP regimen to control HLH and treat Castleman's Disease. When the disease worsened, presenting with respiratory failure, metabolic acidosis, seizures, and hypertension-related encephalopathy, the patient was switched to single-drug treatment with tocilizumab. After 18 courses of chemotherapy, the patient's condition improved compared to before, with no progression or recurrence. Tocilizumab treatment was discontinued after a year and a half, and the patient has been off medication for 28 months without recurrence.

Patient 3 improved after treatment with IL-6 receptor monoclonal antibody.

Patient 9 initially received siltuximab monotherapy outside the hospital with poor results, but improved after switching to tocilizumab monotherapy at our hospital.

Six out of 9 patients switched to other treatment regimens due to poor response to anti-IL-6 treatment.

Patient 1 showed no significant improvement in affected organs such as the liver and spleen after three courses of IL-6 receptor antibody treatment. After a comprehensive evaluation of the patient's condition, steroid monotherapy was administered for 19 weeks. The patient then presented with abdominal pain again and was switched to sirolimus treatment. After 3 months of stable condition, the patient discontinued the medication on their own. One month after discontinuation, the patient's condition progressed again. After 4 weeks of treatment with rituximab, the patient continued to take cyclosporine orally for a year, and the condition was stable at the last follow-up.

Patient 4, who had concurrent POEMS syndrome, initially received IL-6 receptor monoclonal antibody combined with methylprednisolone, but due to progressive neurological symptoms and no significant improvement in systemic inflammatory response, was later treated with lenalidomide combined with dexamethasone. The peripheral neuropathy worsened during the first course of treatment (4 weeks), presenting as muscle weakness; the limb pain improved at the start of the third course; by the ninth month of the course, the muscle strength had significantly recovered compared to before. The patient then switched to single-drug maintenance with lenalidomide for a year. At the last follow-up, the patient had been off medication for 18 months, the muscle strength of the distal and proximal limbs was grade 5, the muscle strength of the distal lower limbs was grade 0, and the condition had improved.

Patient 5 had concurrent TAFRO syndrome, and the pathological subtype was plasma cell type. The patient initially received IL-6 receptor monoclonal antibody combined with methylprednisolone treatment with poor results. After the systemic inflammatory response continued to progress, the patient was switched to BCD regimen (bortezomib, cyclophosphamide, dexamethasone) combined with thalidomide chemotherapy. The total course of treatment was 15 months. At the last follow-up, the patient had been off medication for 15 months, and the condition had improved.Patient 6 was treated with rituximab after poor response to IL-6 receptor antibody treatment outside the hospital. However, there was still an increase in inflammatory indicators. After being admitted to our hospital, the patient was treated with dexamethasone combined with thalidomide, but the patient did not take dexamethasone orally as required. At the last follow-up, the patient had been on treatment for 14 months, IL-6, ESR, CRP levels were slightly elevated, and the condition was stable.

Patient 7 received single-drug treatment with IL-6 receptor monoclonal antibody for 20 months. At the last follow-up, the patient again presented with multiple enlarged lymph nodes in the groin and axilla. A re-biopsy showed no signs of recurrence, and the patient is currently off medication and under observation.

Patient 8 had a poor response to anti-IL-6 treatment before admission, with evident systemic inflammatory response and poor control of multiple serous cavity effusion. After admission to our hospital, the patient was treated with BCD regimen chemotherapy. Currently, the patient is in the third course of medication, and the condition has improved.

## Discussion

Castleman disease is a rare polyclonal lymphoid hyperplasia disease, with proliferative lymph node enlargement as the main feature, and an annual incidence rate of about 2.1/100,000. Pediatric Castleman disease is mostly reported in case reports. Over the past five years, our center has treated 9 children with idiopathic multicentric CD, with a median age of onset of 11 (2–15) years, a wide range of ages at onset, no distinct age characteristics, including 6 males and 3 females. At present, the diagnosis of CD still mainly depends on pathological examination results. UCD often only presents as painless enlargement of a single lymph node, often resulting in missed or misdiagnosis. In terms of pathological typing, previous studies have shown that both UCD and MCD are mainly hyaline vascular type, with 5/9 being hyaline vascular type, which is consistent with previous reports [5, 14, 15].

The pathogenesis of Castleman's disease remains unclear. Studies have shown that overproduction of IL-6 may be one of the mechanisms triggering iMCD [16]. However, IL-6 is not universally elevated in iMCD patients. In this article, 7 out of 9 patients exhibited varying degrees of IL-6 elevation. Simultaneously, retrospective studies have reported that about half of iMCD patients do not respond to IL-6 receptor antibody treatment. In this article, 3 out of 9 patients responded well to anti-IL-6 treatment alone. Studies have indicated that other cytokines and signaling pathways may be involved in the pathogenesis of iMCD, such as vascular endothelial growth factor (VEGF), IL-10, tumor necrosis factor, and γ-interferon [17, 18].

In a small cohort study of children with HHV-8-negative MCD, 67% had systemic symptoms and 75% had elevated laboratory inflammatory markers [5]. Although iMCD has diverse clinical manifestations, lymph node enlargement and related compression symptoms remain the main clinical manifestations. In this article, 6 out of 9 patients mainly presented with lymph node enlargement or related compression symptoms. It is worth noting that pain, limb edema, and other compression-related symptoms are difficult to identify at the beginning of diagnosis and treatment and need to be handled carefully in daily clinical practice. Systemic inflammatory response is also common in iMCD patients, often accompanied by fever, elevated inflammatory indicators, and multi-organ involvement [5, 19, 20]. In pediatric iMCD cases, the degree of elevation of inflammatory marker levels (such as elevated CRP, accelerated ESR) is more apparent than in adults. In this article, 4 out of 9 patients showed varying degrees of CRP elevation, and 2 patients had significantly elevated CRP levels at the onset of the disease, > 100 mg/L; 4 out of 7 patients exhibited elevated ESR. Simultaneously, pediatric iMCD patients are more likely to experience disease progression and recurrence. In this article, 8 out of 9 patients experienced varying degrees of disease recurrence or progression during diagnosis and treatment. However, the overall prognosis is generally good, with few deaths occurring after receiving reasonable treatment [5]. As of the endpoint of follow-up in this article, all patients were alive.

In addition, during the diagnosis and treatment process of Castleman's disease, it is necessary to be alert to the possible severe complications, such as paraneoplastic pemphigus, HLH, TAFRO syndrome, and the variant form of POEMS syndrome in Castleman's disease.

Paraneoplastic pemphigus is an autoimmune syndrome caused by pemphigus concurrent with tumors, which can occur secondary to various neoplastic diseases, including CD. It generally occurs more frequently in UCD, however, there have been reports in the literature that it can also occur secondary to MCD [21]. There have been reports of pediatric cases similar to this overseas [22], but there have been no relevant case reports in China. TAFRO syndrome is a syndrome characterized mainly by thrombocytopenia (T), anasarca (A), fever (F), reticulin fibrosis (R), and organomegaly (O). Previous research considers it closely related to MCD. In this article, Patient 5 was ultimately diagnosed with TAFRO syndrome. So far, there have only been five reports of pediatric TAFRO syndrome overseas [12, 23–26]. In this article, Patient 5, who had concurrent TAFRO syndrome, initially presented with fever and edema in the limbs, progressive thrombocytopenia, and small amounts of effusion in multiple serous cavities. Lymph node biopsy showed typical pathological manifestations of CD. In conjunction with the patient's clinical manifestations and laboratory examinations, all supported the diagnosis of TAFRO [27]. This is also the first report of pediatric TAFRO syndrome in China. Patient 4 presented with peripheral neuropathy, increased λ light chain protein levels in blood electrophoresis results, elevated VEGF levels, organomegaly, effusion in multiple serous cavities, and endocrine disorders such as hypothyroidism and adrenal insufficiency through multiple biochemical tests, all of which are consistent with the clinical features of POEMS. However, literature reports that POEMS syndrome can only be diagnosed when both peripheral neuropathy and monoclonal plasma cells are present in Castleman's disease [28]. In the absence of these conditions but with other clinical features of POEMS syndrome, it can only be diagnosed as a variant form of POEMS syndrome in Castleman's disease. Patient 4 only had elevated λ light chain protein in the blood and lacked evidence of monoclonal plasma cell proliferation, so the final diagnosis was a variant form of POEMS syndrome in Castleman's disease. There are relatively few reports on severe complications related to Castleman's disease in children both domestically and abroad, and this article contributes to helping doctors improve their understanding of the disease.

The generally accepted main treatment for MCD patients is anti-IL-6 treatment, including anti-IL-6 monoclonal antibody treatment and anti-IL-6 receptor monoclonal antibody treatment. Research has found that the clinical application of anti-IL-6 treatment can relieve symptoms and signs [16, 29], and some patients' clinical symptoms improve as IL-6 levels decrease during treatment, indicating that anti-IL-6 treatment can significantly improve the condition of children with CD [25, 30, 31]. Although in UCD patients, the level of IL-6 can also decrease by surgically removing the affected lymph nodes, current research does not advocate routine surgery for MCD children [32]. Patient 3 in this article received anti-IL-6 receptor monoclonal antibody treatment alone and achieved good results. Patient 2, who had concurrent HLH, initially used the CHOP regimen to control HLH and treat Castleman's disease. After the treatment was ineffective, he switched to anti-IL-6 receptor monoclonal antibody single-drug treatment. After 18 courses of treatment, the patient's condition improved compared to before. As of the last follow-up, the patient had stopped taking the drug for 18 months and had not shown any progression or recurrence. Patient 9's condition improved after switching to anti-IL-6 receptor monoclonal antibody treatment from anti-IL-6 monoclonal antibody treatment at an outside hospital. Existing literature reports that the overall effective rate of anti-IL-6 treatment is 34%. Among the 9 patients described in this article, 6 (Patients 1, 4, 5, 6, 7, 8) had suboptimal responses to anti-IL-6 treatment. In the initial stages of high inflammatory response, early application of hormones can quickly control the cytokine storm and can be used in combination with other drugs at the beginning of treatment to control the disease [5, 7, 33]. Due to the heterogeneity of iMCD clinical manifestations, it is necessary to adjust the treatment plan. For patients with severe comorbidities or refractory recurrence, literature reports that immunomodulators such as Anakinra, Bortezomib, Thalidomide, and Sirolimus have been successfully used in some clinical cases [29, 33, 34]. The above-mentioned 6 patients respectively received treatment with Rituximab combined with Cyclosporine, Lenalidomide combined with Dexamethasone, and BCD chemotherapy combined with Thalidomide, all of which achieved relatively good results.

Of the patients we described, 6/9 discontinued their medication at or before the last follow-up visit, with a duration of discontinuation ranging from 0–19 months, and none of them developed significant systemic symptoms and there was no evidence of disease reactivation. This does not mean that their disease has been cured, but the above patients demonstrated a relatively stable period in the course of their disease. In a study of 21 patients with MCD, 71% of the patients maintained sustained remission at 305 days after discontinuation of medication [35]. There is a paucity of studies on the follow-up of MCD after discontinuation of medication, and we will further follow up the children with CD at our center.

## Conclusion

Castleman's disease has a low incidence in children, the exact cause is not yet fully understood, and it is easily confused with other diseases clinically. It is more likely to manifest as severe systemic symptoms. For patients who are highly suspected of having this disease, lymph node biopsy should be completed as soon as possible to avoid missed diagnosis and misdiagnosis. The treatment of CD has no unified standard at present. It is generally recognized internationally to treat iMCD children mainly with IL-6(/receptor) monoclonal antibody. However, for some children with more apparent systemic symptoms or poor response to single-drug treatment with IL-6 receptor monoclonal antibody, there are currently no clear international guidelines. Taking combined chemotherapy or second-line treatment drugs such as Lenalidomide, Thalidomide or Sirolimus, etc., according to the treatment plan for adult iMCD may achieve better results.

## Data Availability

Due to the requirements of the Ethics Committee of Capital Medical University, the datasets generated and/or analyzed during the current research period are not publicly available, but can be obtained from the corresponding authors upon reasonable request.

## Abbreviations

iMCD: Idiopathic multicenter Castleman disease
HLH: Hemophagocytic lymphohistiocytosis
IL-6: Interleukin-6
HHV-8: Human herpesvirus-8
VEGF: Vascular endothelial growth factor
CRP: C-reactive protein
ESR: Erythrocyte sedimentation rate
